# Review and comparison of clinical evidence submitted to support European Medicines Agency market authorization of orphan-designated oncological treatments

**DOI:** 10.1186/s13023-015-0349-z

**Published:** 2015-10-28

**Authors:** Julie Winstone, Shkun Chadda, Stephen Ralston, Peter Sajosi

**Affiliations:** SIRIUS Market Access, 58 St Kilda Rd, London, W13 UK; Takeda Pharmaceuticals International GmbH, Zurich, Switzerland

**Keywords:** European Medicines Agency, Clinical trials, Mifamurtide, Orphan disorders, Orphan treatments, Osteosarcoma

## Abstract

**Background:**

Clinical trials for treatments indicated for orphan diseases may be limited due to the low prevalence of such diseases; this can result in implications for both regulatory and health economic perspectives. This study assessed the pivotal clinical evidence packages submitted to support applications for European Medicines Agency (EMA) marketing authorizations for treatments for orphan conditions, in relation to the size of the eligible patient population.

**Methods:**

Approved treatments for EMA-designated orphan conditions (defined as life-threatening or chronically debilitating conditions that affect ≤5/10,000 people) were identified from the EMA web site. All treatments reviewed were included in anatomical therapeutic chemical (ATC) category L (antineoplastic and immunomodulating drugs): this category was selected because it is the largest ATC category, containing almost 50 % of all approved orphan-designated products. Treatments were reviewed if they had been approved within the past 7 years and had been evaluated in a controlled trial using at least one survival-based clinical endpoint. Treatments were compared in terms of patient-years (accumulated duration of follow-up), the number of patients in the pivotal trials and disease prevalence.

**Results:**

As of 1 February 2014, 68 treatments had been approved for orphan-designated conditions, of which 30 belonged to ATC category L and 14 met all inclusion criteria. The number of patients in the pivotal trials ranged from 162 to 846 (median 485). In terms of patient-years, the longest duration of follow-up was seen in the pivotal trial of mifamurtide in osteosarcoma, which had 4068 patient-years; excluding this trial, follow-up ranged from 308 to 2906 patient-years (median 1796 years). Osteosarcoma had the second smallest eligible patient population (0.5/10,000 persons) of the reviewed treatments.

**Conclusions:**

Clinical trials of orphan treatments are often limited by low patient numbers and inadequate follow-up. Pooling of expertise in single centres and the establishment of rare disease reference networks and patient registries may facilitate appropriate trial design for orphan-designated treatments. This analysis found that the pivotal clinical trial for mifamurtide in osteosarcoma had the largest number of patient-years of follow-up, despite a small eligible patient population, showing that it is possible to conduct studies with an adequate patient population size and duration of follow-up in patient-years, and a comparative design with clinical, survival-based, endpoints.

## Background

Although definitions of orphan diseases vary, it is generally accepted in most countries that such disorders affect between 1 and 8 individuals per 10,000. Within the European Union (EU), orphan conditions are defined by the European Medicines Agency (EMA) as life-threatening or chronically debilitating conditions that affect no more than 5 in 10,000 people (equivalent to approximately 250,000 or fewer people for each condition) in the EU [1–3].

There are difficulties in balancing the urgent need for drugs to treat rare diseases with the requirements for guaranteed quality, efficacy and safety and, when necessary, making comparisons with existing therapies. Reliable methods of evaluating drugs in small numbers of patients are problematic, adding to difficulties in producing high-quality dossiers, despite incentives for pharmaceutical companies to develop such products and the use of less stringent criteria for trials of drugs with designated orphan indications [4]. These problems are evident even when the efficacy of a potential treatment is well established in other indications [1]. For these reasons, clinical data submitted to support an application for marketing authorization of treatments for orphan conditions may often be less robust than is the case for treatments for non-orphan common conditions. In a study in 2006, for example, 10 of 18 (55 %) approved orphan-designated treatments were authorized ‘under exceptional circumstances’, indicating that the clinical dossiers were incomplete and that further studies would be needed to maintain the marketing authorization [4]. Overall, only nine of the approved treatments (50 %) were supported by RCT data: five were supported only by uncontrolled Phase II studies, two by uncontrolled open-label studies, and one by a literature analysis alone. Other limitations of the submitted data included the use of surrogate endpoints and inadequate durations of follow-up in relation to the natural history of the disease. A subsequent study by the same authors suggested that this situation persisted throughout the first decade after implementation of orphan drug legislation in the EU [5].

The limitations of the clinical evidence submitted to support marketing authorizations for orphan treatments have a number of implications. Poorly designed pivotal trials may contribute to a low rate of success for such applications both in the EU [4, 6] and the USA [7]. Furthermore, limited pivotal trial data, together with the high cost of many orphan treatments, may create difficulties in terms of reimbursement and market access [1, 8, 9]. As the number of orphan-designated treatments entering clinical practice increases, it is possible that the data supporting an application for marketing authorization may increase. Indeed, it has recently been suggested that submission of objective data on the natural history of a disease and the potential impact of the proposed new treatment could form an additional criterion for decision-making on market access and reimbursement policies [10]. A further consideration is that marketing authorizations based on limited clinical data could potentially expose the patient to increased risk of adverse events or lack of efficacy, compared with non-orphan treatments [11]. In view of these issues, the present study was performed to evaluate the pivotal clinical evidence packages presented in the European Public Assessment Report for orphan-designated treatments with an EMA marketing authorization.

## Methods

This study reviewed the clinical evidence packages for treatments approved by the EMA. All treatments included in the review belong to anatomical therapeutic chemical (ATC) category L (antineoplastic and immunomodulating agents); this category was chosen because it is the largest ATC category, containing almost 50 % of all orphan-designated treatments that have received marketing authorization from the EMA. Treatments were eligible for inclusion in the review if they had been approved after 31 December 2006 and had been evaluated in a comparative trial using at least one survival-based clinical endpoint as opposed to short-term surrogate endpoints. The time limit was imposed to ensure that all included treatments had been evaluated by the EMA using similar criteria: it is possible that older treatments might have been evaluated using less stringent criteria than recently approved treatments. Similarly, the requirement for comparative studies using survival-based endpoints was imposed because such studies can be considered the ‘gold standard’ for clinical trials in oncology and are recommended in United States Food and Drug Administration guidance [12]. Eligible treatments were compared in terms of the number of patients included in pivotal (‘main’) clinical trials submitted to support the application for marketing authorization, the cumulative duration of follow-up in the pivotal trials, expressed in patient-years, and the prevalence of the orphan condition. The number of patient-years of follow-up was calculated by multiplying the total number of patients in each study (across all treatment arms) by the study duration; where the study duration was not reported explicitly, Kaplan-Meier curves or data tables were used to estimate the duration of follow-up. Prevalence data for each orphan-designated condition were obtained from the relevant EMA Committee for Orphan Products public summary of opinion [3]: where these data were provided as the number of affected patients in the EU, this was converted to the rate per 10,000 based on the EU population at the time of orphan designation.

In addition to patient numbers and duration of follow-up, the quality of the pivotal trials for all orphan products approved by the EMA since 31 December 2006 was assessed by means of the Jadad scoring system [13]. In this system, a maximum of two points each are given for appropriate randomization and blinding, and a further point is given if all patients are adequately accounted for; thus, the maximum possible Jadad score is 5.

## Results

As of 1 February 2014, 68 treatments with 87 indications had received an EMA marketing authorization for orphan-designated conditions. The prevalence of the orphan indications varied markedly, from 0.00125/10,000 persons for N-acetylglutamate synthetase deficiency to <4.5/10,000 persons for adrenal insufficiency.

Of the 68 orphan treatments, 30 belonged to ATC category L (antineoplastic and immunomodulating agents); of these, 21 had received a normal marketing authorization, four had received a conditional authorization, and five were authorized under exceptional circumstances. The 30 treatments were approved for 41 orphan indications: 19 were approved for a single indication and 11 were approved for two indications. Four treatments were excluded from the analysis because only literature reports, rather than clinical trial data, were provided as pivotal study evidence. For the remaining 37 indications, 52 % of the pivotal studies were Phase III trials, and randomized clinical trial (RCT) data were provided in 57 %. Overall, 73 % of studies had at least one clinical endpoint, but only seven studies (15 %) included a survival-based clinical primary endpoint.

**Fig. 1:**
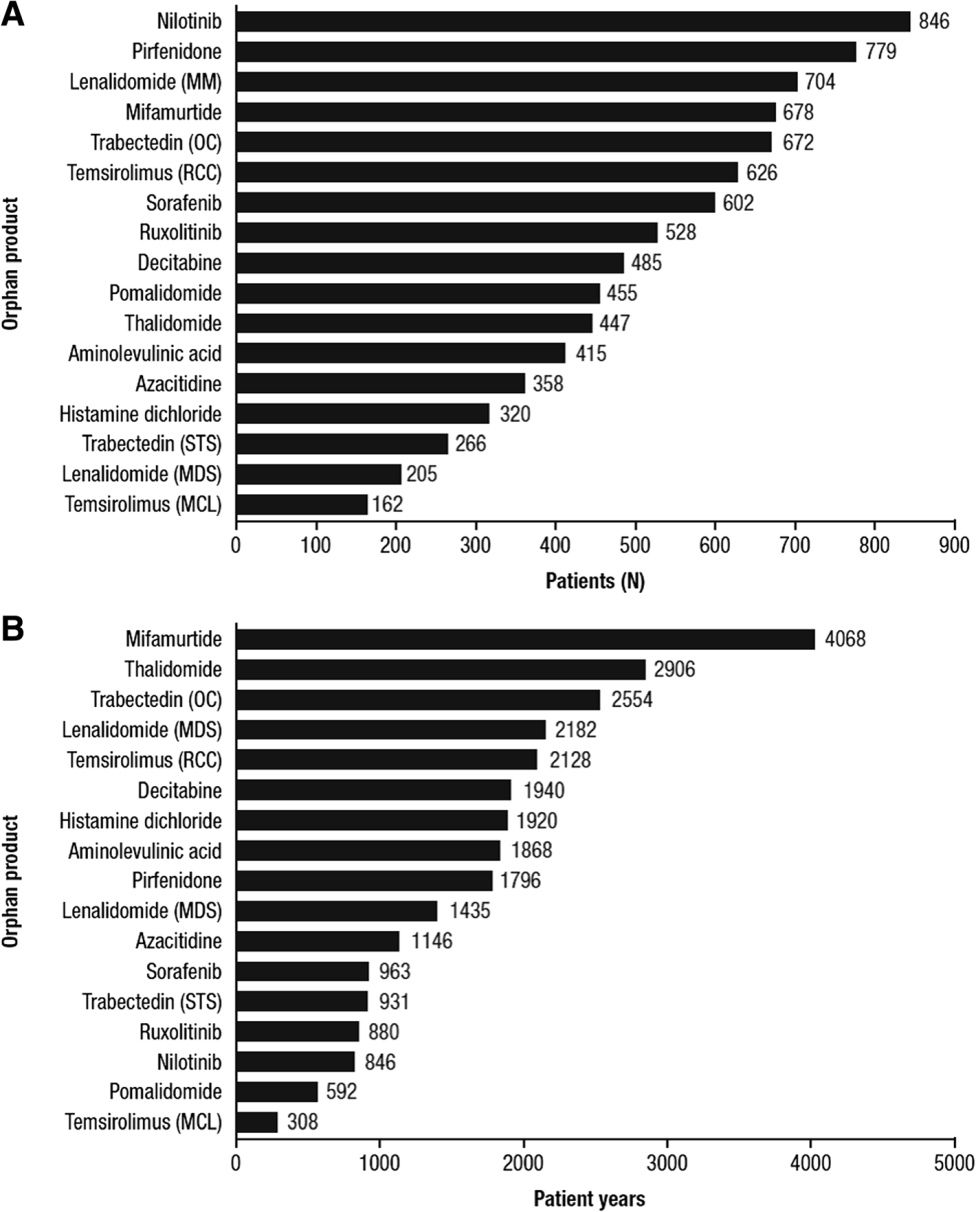
Number of patients (**a**) and cumulative duration of follow-up (**b**) in included studies. All treatments belonged to ATC class L (antineoplastic and immunomodulatory agents). For inclusion in the review, treatments were required to have received EMA authorization for orphan-designated conditions within the previous 7 years, and to be supported by comparative trials with at least one survival-based clinical endpoint. Duration of follow-up was estimated by multiplying the total number of patients in each study by the study duration, or from Kaplan-Meier curves if these data were not available. MCL, mantle cell lymphoma; MDS, myelodysplastic syndrome; MM, multiple myeloma; OC, ovarian cancer; RCC, renal cell carcinoma; STS, soft tissue sarcoma

**Fig. 2:**
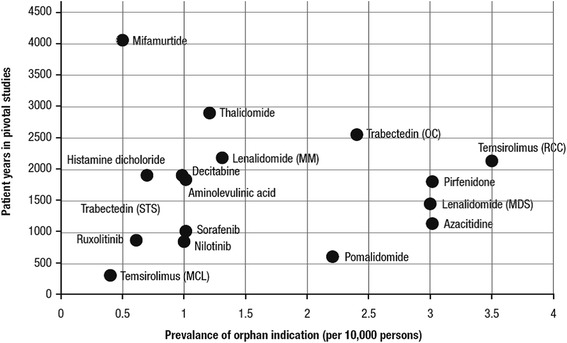
Cumulative duration of follow-up in relation to the reported prevalence of the orphan conditions. Prevalence data derived from the relevant EMA Committee for Orphan Medicinal Products public summary of opinion for each treatment [3]. EMA, European Medicines Agency; MCL, mantle cell lymphoma; MDS, myelodysplastic syndrome; MM, multiple myeloma; OC, ovarian cancer; RCC, renal cell carcinoma; STS, soft tissue sarcoma

A total of 14 products met all the inclusion criteria, having been authorized within the previous 7 years and supported by comparative trial data with at least one survival-based clinical endpoint (Table 1). The median number of patients included in the pivotal trials of these treatments was 485, and the number of patients ranged from 162 in a trial of temsirolimus in mantle cell lymphoma to 846 in a trial of nilotinib in chronic myelogenous leukaemia (Fig. 1a). The cumulative duration of follow-up, expressed in patient-years, also varied widely (Fig. 1b), from 308 patient-years in the temsirolimus trial in mantle cell lymphoma to 4068 patient-years in a trial of mifamurtide in osteosarcoma [14]. Follow-up in the latter trial appeared to be particularly extensive: when this trial was excluded from the analysis, the duration of follow-up ranged from 308 to 2906 patient-years, with a median of 1796 patient-years. A comparison of the duration of follow-up with the reported prevalence of the orphan condition (Fig. 2) showed that the osteosarcoma indication for mifamurtide, at 0.5/10,000, had the second smallest eligible patient population of the reviewed treatments: only the mantle cell lymphoma indication for temsirolimus had a lower prevalence (0.4/10,000).

**Table 1:** Orphan-designated treatments meeting criteria for review

Generic name	Proprietary name	Orphan condition	Prevalence of orphan condition
Histamine dichloride	Ceplene	Adult acute myeloid leukaemia in first remission	0.7/10,000
Decitabine	Dacogen	Adult acute myeloid leukaemia	<1/10,000
Pirfenidone	Esbriet	Adult mild to moderate idiopathic pulmonary fibrosis	≤3/10,000
5-aminolevulinic acid	Gliolan	Visualization of malignant tissue during surgery for malignant glioma in adults	1/10,000
Pomalidomide	Imnovid	Multiple myeloma	2.2/10,000
Ruxolitinib	Jakavi	Splenomegaly or symptoms in adult patients with primary myelofibrosis	0.5/10,000
Mifamurtide	Mepact	Osteosarcoma	0.5/10,000
Sorafenib	Nexavar	Hepatocellular carcinoma; renal cell carcinoma	Hepatocellular carcinoma: 1/10,000; renal cell carcinoma: not reported
Lenalidomide	Revlimid	Multiple myeloma	1.3/10,000
Nilotinib	Tasigna	Adult patients with newly diagnosed Philadelphia-chromosome-positive chronic myelogenous leukaemia	46,000 persons in EU (calculated as 1.0/10,000 based on EU population 457.7 million)
		Adult patients with Philadelphia-chromosome-positive chronic myelogenous leukaemia with resistance or intolerance to prior therapy including imatinib	
Thalidomide	Thalidomide Celgene	Untreated multiple myeloma in adults aged > 65 years or ineligible for high-dose chemotherapy	1.2/10,000
Temsirolimus	Torisel	Renal cell carcinoma; adult patients with relapsed and/or refractory mantle cell lymphoma	Renal cell carcinoma: 3.5/10,000; refractory mantle cell lymphoma: 0.4/10,000
Azacitidine	Vidaza	Adults not eligible for haematopoietic transplant with myelodysplastic syndromes, chronic myelogenous leukaemia, and acute myeloid leukaemia	1.1–3/10,000
Trabectedin	Yondelis	Treatment of patients with advanced soft tissue sarcoma	23,000 persons in EU (calculated as 0.6/10,000 based on EU population 377 million)

*EU* European Union

In general, the quality of the pivotal trials, as assessed by Jadad scores, was moderate. The mean (±SD) Jadad score was 2.6 ± 1.8, and the median score was 3 (range 0–5). Of the 116 trials assessed, only 74 (63.8 %) were randomized and 55 (47.4 %) were blinded; the numbers of trials considered adequately randomized or blinded were 41 (35.3 %) and 45 (38.8 %), respectively. The trial with the most extensive follow-up, the mifamurtide osteosarcoma trial, had an overall Jadad score of 3, reflecting its randomized open-label design.

## Discussion

The results of this study show that pivotal trials of treatments for orphan conditions are often limited by a number of methodological factors, including a lack of randomization or blinding, low patient numbers (median 485) and limited follow-up (median 1796 patient-years). It is important to emphasize however that, notwithstanding these limitations, all the treatments were approved by the EMA.

These findings are consistent with previous studies that have found significant limitations in the design of clinical trials for orphan treatments both in the EU [4, 5] and the USA [7]. Factors contributing to these limitations include a lack of pooling of manpower and resources for rare diseases. The lack of specialist centres with multidisciplinary teams with expertise in particular rare diseases can result in a lack of data to understand the natural history of the disease or to inform clinical and transitional research. This situation may be improved by the establishment of sustainable patient registries for specific rare diseases and European reference networks of patient registries [15], which should facilitate active and prospective monitoring of clinical interventions for rare diseases [16].

Orphan drug policies may be unsatisfactory from several viewpoints. For example, access to orphan drugs may be restricted in patients with some rare diseases, and funds may not be made available to pay for therapies when they have been developed [17]. Against this background, the low prevalence of orphan disorders (<5/10,000 population in the EU) remains a major problem in the design of pivotal clinical trials [4]. Even when the prevalence is relatively high compared with other orphan conditions (as is the case for many of the products included in this review), trials may still be conducted in an inadequate number of patients. A study by Joppi et al., for example, found that pivotal trials in treatments for Fabry disease included only 41 and 56 patients out of a potential patient population of 10,000 in Europe [4]. By contrast, the present study found evidence that a small patient population need not necessarily be a barrier to adequate patient recruitment. The pivotal study of mifamurtide in osteosarcoma had the largest number of patient-years of follow-up and the fourth largest number of patients of all reviewed treatments, despite the low prevalence of osteosarcoma (0.5/10,000). Indeed, it is noteworthy that only one indication, for temsirolimus in mantle cell lymphoma, had a smaller eligible patient population (Fig. 2); this pivotal trial also had the smallest number of patients and the shortest cumulative follow-up. Three pivotal trials, for pirfenidone in idiopathic pulmonary fibrosis, lenalidomide in multiple myeloma, and nilotinib in chronic myeloid leukaemia, included more patients than the mifamurtide trial; however, in each case the prevalence of the orphan condition was higher than that of osteosarcoma (≤3/10,000, 1.3/10,000 and 1.0/10,000, respectively).

Although this analysis has focused on patient numbers and duration of follow-up as a measure of trial quality, a number of other potential measures are available, including the Jadad scoring system [13] and the criteria of the Oxford Centre for Evidence-based Medicine [18] and the Cochrane Collaboration [19]. Application of the Jadad system to the studies included in this analysis confirmed that the trials had significant limitations with respect to randomization or blinding, as discussed below.

In addition to inadequate patient numbers and follow-up, pivotal trials of orphan treatments may also be limited by the use of surrogate endpoints rather than clinical survival-based endpoints. In the present study, five of the 30 ATC category L treatments were excluded from the review for this reason. Although desirable, the use of survival-based clinical endpoints may make it difficult to demonstrate statistically significant treatment effects in trials of orphan treatments where, as noted above, patient numbers and duration of treatment may be limited [7, 20]. Conversely, it may be easier to demonstrate significant treatment effects on surrogate endpoints, but such endpoints are less likely to be acceptable to regulators [7, 21]. The choice of endpoint therefore reflects a balance between the need for adequate statistical power in the trial and the requirements of regulatory authorities [7].

A further issue is the lack of randomization or blinding, or both, in many pivotal trials of drugs for orphan conditions. It is noteworthy that more than a third of the trials reviewed in the present study were non-randomized, and less than 40 % were appropriately randomized or blinded. Indeed, the three trials with the most extensive follow-up in terms of patient-years (mifamurtide in osteosarcoma, thalidomide in untreated multiple myeloma, and trabectedin in ovarian cancer) all had randomized open-label designs. In many cases, the lack of randomization may reflect the small number of patients with the condition; there are also ethical considerations relating to the use of placebos in life-threatening conditions for which no alternative therapy is available for use as a comparator.

In the present study, treatments were only eligible for review if they had received EMA approval after 31 December 2006, in order to maximize the likelihood that all reviewed treatments had been evaluated according to similar criteria. The finding that methodological issues relating to pivotal trials of the approved products were similar to those reported by Joppi et al. in 2006 [4] suggests that the criteria for evaluation of orphan treatments have not become more stringent over time. A recent study, which reviewed the EMA evaluations of 17 orphan drugs and 51 non-orphan drugs conducted during 2009–2010, concluded that the regulatory standards applied to orphan drugs were as rigorous as those applied to non-orphan drugs [22].

## Conclusions

The results of this analysis show that 30 treatments belonging to ATC category L (antineoplastic and immunomodulating agents) received an EMA marketing authorization for 41 orphan indications between 31 December 2006 and 1 February 2014. Of the 37 indications for which clinical trial data were presented, 52 % of the pivotal studies were Phase III trials and RCT data were provided in 57 % of the pivotal studies. Because this analysis was restricted to ATC category L products, and the prevalence of oncological orphan diseases is often higher than that of many non-oncological orphan diseases, the results may not be applicable to the latter situation.

This study shows that problems relating to low patient numbers, or other methodological limitations, do not necessarily preclude approval of an orphan product. The pivotal clinical trial for mifamurtide in osteosarcoma had the largest number of patient-years of follow-up (4068 patient-years) of any of the reviewed treatments, despite a small eligible patient population (0.5/10,000 persons); by contrast, the next largest study, of thalidomide in multiple myeloma, had 2906 patient-years of follow-up in an eligible population of 1.2/10,000 persons. Furthermore, each of the three treatments with the longest durations of follow-up were limited by a lack of blinding or (in the case of the thalidomide and trabectedin trials) the use of suboptimal endpoints. Nevertheless, each of these trials was considered adequate to support approval of the treatment by the EMA.

The low prevalence of orphan diseases is an important factor in the limited patient numbers and inadequate follow-up in many of the clinical trials included in the dossiers. Pooling of expertise through the establishment of rare disease reference networks and patient registries may improve this situation.

## Abbreviations

ATC: Anatomical therapeutic chemical
EMA: European Medicines Agency
EU: European Union
RCT: Randomized controlled trial
